# Classical Dichotomy of Macrophages and Alternative Activation Models Proposed with Technological Progress

**DOI:** 10.1155/2021/9910596

**Published:** 2021-10-21

**Authors:** Yali Wei, Mengxi Wang, Yuwen Ma, Zhenni Que, Dengbo Yao

**Affiliations:** ^1^Department of Oral Implantology & National Clinical Research Center for Oral Diseases & State Key Laboratory of Oral Diseases, West China Hospital of Stomatology, Sichuan University, Chengdu, China; ^2^Minhang Branch, Zhong Shan Hospital, Fudan University, China

## Abstract

Macrophages are important immune cells that participate in the regulation of inflammation in implant dentistry, and their activation/polarization state is considered to be the basis for their functions. The classic dichotomy activation model is commonly accepted, however, due to the discovery of macrophage heterogeneity and more functional and iconic exploration at different technologies; some studies have discovered the shortcomings of the dichotomy model and have put forward the concept of alternative activation models through the application of advanced technologies such as cytometry by time-of-flight (CyTOF), single-cell RNA-seq (scRNA-seq), and hyperspectral image (HSI). These alternative models have great potential to help macrophages divide phenotypes and functional genes.

## 1. Introduction

Macrophages are an important part of the immune system and can secrete cytokines and growth factors to regulate the occurrence and development of inflammation and can transform their phenotype under a variety of different stimuli which is called activation or polarization [1–3]. The regulation of macrophage activation has become important in immunology [4]. The classic macrophage dichotomy activation model divides macrophages into M1 and M2 *in vitro* based on the type of stimulation, surface molecules, secreted cytokines patterns, and functional characteristics [5, 6]. However, the stimulation of macrophages in the in vivo environment is more complicated than *in vitro* experiments and, due to the emergence of macrophage heterogeneity, shows the limitations of the classic activation dichotomy. In recent years, more information about the behavior of macrophages in diseases and tissue-specific phenotypes has been obtained through different technologies, and some scholars have proposed alternative macrophage activation models, such as comprehensive multidimensional models and spectral models. Alternative classification methods derived using advanced technical methods provide the potential to identify phenotypes and molecular markers associated with specific disease characteristics associated with macrophages.

The purpose of this study is to clarify the argument of the classic dichotomy and introduce different macrophage activation models that have been proposed due to advanced technologies, so that researchers can better classify macrophages and provide a theoretical basis for interventional therapy targeting specific biomarkers of macrophages.

## 2. Classical Dichotomy Model of Macrophages

### 2.1. Development of Dichotomy Model

The proposal and development of the dichotomy model have been supplemented by numerous studies. The earliest macrophage activation model described the behavior and gene expression changes of macrophages stimulated by interleukin 4 (IL-4) as “selective activation,” while macrophages stimulated by interferon-g (IFN-g) were described as “classic activation” [7]. Later, Mills et al. [8] put forward the concept of M1/M2 dichotomy based on the difference of arginine metabolism between macrophages from C57BL/6 and macrophages from Balb/c mice. They believed that M1/M2 was the inherent attribute of macrophages in the transition from inflammation to healing, which occurred in the absence of adaptive immune response, and appeared in the early stages of evolution [9]. According to different activation scenarios and combined with the results of spectral analysis, the dichotomy has been further developed, and M2a, M2b, and M2c have been proposed [10]. Then, Fleetwood et al. [11] observed significant differences in the transcriptional expression of colony-stimulating factor 1 (CSF-1) and granulocyte-macrophage colony-stimulating factor (GM-CSF) after growth, and they described the macrophages growing in GM-CSF as M1 and the macrophages in CSF-1 as M2 [12].

### 2.2. The Main Characteristics of M1/M2 Macrophages Proposed by the Dichotomy Model

In the classical dichotomy model, the phenotype of macrophages is determined by the environmental signal network. According to different types of stimulation, surface molecules, secreted cytokines, and functional characteristics, activated macrophages are divided into two phenotypes: classically activated macrophages (M1) and alternately activated macrophages (M2) [13–16]. M1 macrophages, also known as classically activated macrophages, can be activated by bacterial lipopolysaccharide (LPS), interferon-gamma (IFN-*γ*), GM-CSF, or tumor necrosis factor (TNF) [17–19]. M1 macrophages are characterized by high expression of proinflammatory cytokines, such as interleukin 12 (IL-12), interleukin 23 (IL-23), TNF-*α*, IL-1*α*, IL-1*β*, IL-6, cyclooxygenase-2 (COX-2), and low expression of interleukin 10 (IL-10), and they have robust antimicrobial and antitumoral activity, mediate ROS-induced tissue damage, impair tissue regeneration, and promote TH1 response and wound healing [20–22]. M2 macrophages, also called alternately activated macrophages, can be further divided into four phenotypes: M2a, M2b, M2c, and M2d [23–26]. M2a, named wound-healing macrophages, can be activated by IL-4 or interleukin 13 (IL-13); M2b can be activated upon combined exposure to immune complexes (IC) and Toll-like receptor (TLR) agonists or by IL-1R agonists; M2c, called inactivated macrophages, can be activated by transforming growth factor-*β* (TGF-*β*) and cortex hormones; and M2d, known as tumor-associated macrophages (TAMs), can be activated by costimulation with TLR ligands and A2 adenosine receptor (A2R) agonists or by IL-6 [27, 28]. M2 macrophages have the functions of immune regulation, anti-inflammation, promoting wound repair, angiogenesis, and resisting the growth of parasites and tumors [29, 30] . And they have the characteristics of high IL-10, low IL-12, and high IL-1 decoy receptor phenotype [25, 31–33].

### 2.3. Macrophages in Implant Dentistry

Macrophages are the principal cells in the innate immune reactions to implants. When the biomaterial is implanted into the host, the host will active a foreign body reaction (FBR), and the FBR can regulate the tissue repair of the implanted site by releasing of damage-associated molecular patterns from the injury to the implant site and to the material itself [34, 35]. By changing the characteristics of the implant, the effect of host immune response can be regulated, and then tissue repair can be promoted [2]. Hotchkiss et al. [36, 37] have shown that macrophages are particularly important to this response, ultimately driving the conclusion of the inflammatory phase and recruiting mesenchymal stem cells (MSCs) to begin the reparative phase or recruiting other inflammatory cells to delay the healing response [38, 39]. In fact, the polarized subtypes of macrophages have no certain advantages and disadvantages to tissue repair; for example, the formation of the vascular network can promote bone tissue regeneration, while the initiation of angiogenesis depends on M1 macrophages, while M2 macrophages play a role in promoting angiogenesis [40–42]. In addition, a too long polarization period of M1 macrophages will lead to an increase in the number of M2 macrophages, resulting in increased secretion of fibronectin, resulting in fiber wrapping on the surface of the implant and affecting the attachment of osteocytes to the surface of the implant [43–45]. Also, they have an important role in the osseointegration of implants to the host recipient and determine the success of the implant [46, 47].

## 3. Shortcomings of the Classic Dichotomy of Macrophages

The classical M1/M2 dichotomy model based on *in vitro* provides a conceptual framework for describing the activation of macrophages in vivo and identifying the corresponding stimuli [13]. However, a large amount of research data shows that the classical dichotomy model is too extreme to reflect the whole process of macrophage activation. Due to a large number of stimuli in the environment and the interaction between stimuli, the spectrum of tissue macrophages will show complexity and overlap [7, 28]. Recent researches show that the performance of macrophages under certain special conditions is not representative, the cell surface markers may be contradictory, and their phenotype may change over time during the course of the disease. These researches further illustrate the limitations of the dichotomy.

In some special stages, such as embryonic macrophages, digestive macrophages, and macrophages from certain cancers, macrophages did not show a representative M1 or M2 phenotype [48]. Stables et al. [49] used zymosan to induce digestive macrophages from peritonitis and compared them with M1/M2 macrophages derived in the vitreous. The results showed that the digested macrophages were neither classically activated nor alternately activated but had certain characteristics of the two phenotypes [49].

In previous studies, M1/M2 macrophages could be distinguished by unique markers expressed on the cell surface, but many studies have shown that this classification method is contradictory. Chang et al. [31] used scRNA-seq to analyze macrophages from the aorta. They divided macrophages into three clusters: inflammatory, resident-like, and a different type of macrophages that highly expressed the triggering receptor expressed on myeloid cells 2 (TREM2). Among them, inflammatory macrophages highly expressed M1-related genes, such as IL-1, TNF, and CXC chemokine ligand 10 (CXCL10), and resident-like macrophages expressed M2 genes, such as mouse macrophage mannose receptor 1 (MRC1), folate receptor 2 (FOLR2), and homo sapiens coagulation factor XIII A1 polypeptide (F13A1). However, MRC1, which encodes the mannose receptor CD206, is usually used to define M2 macrophages and was also expressed in a subset of inflammatory macrophages [50]. Helm et al. [51] performed a phenotypic analysis of tumor-associated macrophages derived from pancreatic ductal adenocarcinoma and found that tumor-associated macrophages also showed M1 (human leukocyte histocompatibility antigen-DR, IL-1, or TNF-*α*) and M2 (mannose receptor CD163 and IL-10) characteristics. These experiments show that macrophages can exhibit anti-inflammatory and proinflammatory properties at the same time. Therefore, the traditional classification of macrophages into M1 and M2 phenotypes cannot fully reflect the diversity of the in vivo population [52].

Macrophages are markedly plastic cells that can transform from one phenotype to another [53]. For example, in the case of myocardial infarction, allergic skin, and skeletal muscle damage, the phenotype of macrophages changed as the disease progresses [48]. Arnold et al. [54] studied the phenotype and function of skeletal muscle monocytes/macrophages during the repair process. In *in vitro* experiments, injured skeletal muscle recruited proinflammatory macrophages for phagocytosis, and then, the proinflammatory macrophages were rapidly transformed into anti-inflammatory macrophages, thereby stimulating muscle generation and fiber growth [54]. The above experiments prove that the activation state of macrophages is not always the same, and it can be reciprocally transformed under some peculiar stimuli.

## 4. Different Activation Models

Because the classical dichotomy M1/M2 model cannot describe the activation of macrophages sufficiently, some scholars have proposed different models of macrophage activation in recent years.

### 4.1. Active Comprehensive Multidimensional Model

The comprehensive multidimensional model of activation integrates signals that act on the specific microenvironment of macrophages and presents a multidimensional view of macrophage activation. The core view of this model is the interaction of stress signals caused by ontogeny, local tissue microenvironment, and tissue damage to stimulate the activation of macrophages [48]. Studies have shown that the same stress signal and the same dynamics will cause different sources of macrophages to produce different results, and the first stress signal will also affect the response of macrophages to the stress signal at a later point in time [48]. Schultze [55] generated a mass transcriptome data set from human macrophages activated by several different stimuli and used mathematical and bioinformatics methods to compare the dichotomous model with the multidimensional model. Through discrete stimulation of 29 human macrophages, the results showed that macrophages respond by a signal input to a specific functional program and combined with the experiments of Gosselin et al. [56], they demonstrated that environmental signals shaped the functional program of macrophages. Therefore, the multidimensional model can be used to evaluate macrophages from different tissues in other disease environments, and the multidimensional model can better reflect the activation state of macrophages than the dichotomy model. Besides, they also found that a large number of transcriptional regulators exhibited transcriptional changes when providing different stimuli to human macrophages, which reflects a subtle transcriptional regulatory network in response to exogenous stimuli [56].

As an extension of the multidimensional model, some scholars based on *in vitro* controlled experiments to find the precorrelation between stimuli and gene expression readings and proposed a stimulus-specific naming system for macrophage activation. The naming of macrophages stimulated outside the receptor will be designated by the inducing stimulus they receive, such as M (LPS). In vivo-derived macrophages will be described by multiple markers, rather than directly categorizing them as M1 or M2. It can be seen that the history of macrophage activation research has evolved from a dichotomy model to a more precise system linking stimuli and phenotypes. The current challenge is to expand the phenotypic classification of macrophages to reflect their functions at specific time points and environments [12, 48].

### 4.2. Spectral Model

**Figure 1:**
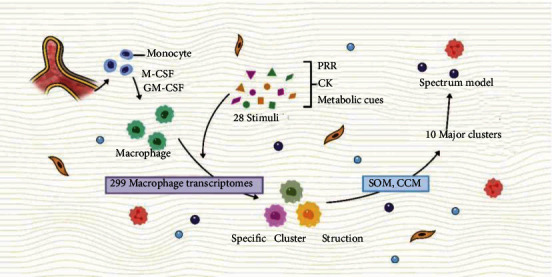
Spectral model. Monocytes are transformed into macrophages by stimulation of M-CSF or GM-CSF. Through 28 stimuli from PRR, cytokines, metabolic cues, etc., 299 macrophage transcription programs can be obtained. The results of the study confirm that each stimulus can correspond to a particular structural cluster. Through the analysis of SOM and CCM, 10 main clusters can be summarized, which is the spectral model.

The researchers used different activation signals to stimulate human macrophages and obtained a data set of 299 macrophage transcripts. They compared and analyzed different stimuli set on a single microarray platform under highly standardized conditions, thereby revealing the spectrum of macrophage activation states, and through network analysis, they identified all-important transcriptional regulators associated with macrophage activating factors, as well as those associated with stimulating specific programs. Finally, they performed network modeling on this data set and expanded the current M1/M2 model to a “spectral model” with at least nine different macrophage activation programs [57]. The researchers mainly analyzed the transcription process of macrophages activated by 28 different stimuli (such as pattern recognition receptor ligands, cytokines, and metabolic chains). Through coregulation analysis (CRA), the overall relationship between these activation state data was confirmed: the activation data state forms a virtual axis where the macrophages at the baseline were placed between the M1 and M2 macrophages stimulated by INF-*γ* and IL-4, respectively. When other conditions related to M1 or M2 activation were added, the overall M1 and M2 axes did not change, and when stimuli that were not related to M1 or M2 activation were added, the macrophage activation signal spectrum outside the initial bipolar axis became obvious. Besides, samples generated by adding early stimulation points showed that the spectrum of macrophage activation was composed of a dense network of individual characteristics. Finally, by using the coordinates of the CRA-defined sample in the 10 clusters defined by the correlation coefficient matrix to construct the vector sum in the three-dimensional space, the macrophage activation model was described by the transcription program profile (the spectrum of the macrophage activation model) [57].  Figure 1 is shown as follows.

### 4.3. Other Views about Macrophage Activation Model

Villani et al. [58] researched the use of CSF to differentiate peripheral monocytes into macrophages (baseline macrophages), then they used conventional differentiation protocols and various standard stimuli for stimulation. Each stimulus condition resulted in a specific activated macrophage phenotype, and CyTOF was used to compare different macrophage phenotypes with baseline macrophages, finally determining the phenotypic pattern reflecting each different activation state. For example, LPS-induced macrophages were characterized by high levels of CD13 and CD86 and low levels of CD163 and CD206. IL-4 induced differentiation of macrophages with high CD274 and low CD64. IFN-g induced macrophages with high CD64 and CD86, while IL-10 induced macrophages with high expression of CD14, CCR2, and CD163. IL-6 induced differentiation of macrophages with high CD11c and high CD33 [11, 58]. Murray et al. [12] believed that macrophages did not form a stable subpopulation but responded to a variety of factors existing in the tissue. And they thought the M1/M2 dichotomy model was usually related to the characteristics of mature macrophages, and activation should occur in an expanded macrophage family which included monocytes, myeloid-derived dendritic cells, and multinucleated giant cells. In the organization, all the links were combined to produce a resulting phenotype, and no single hierarchical structure or sequence could represent the biological characteristics of the cell. Therefore, while studying the activation model, it is necessary to dynamically observe this process to consider the various elements in its whole body and local environment and to define the dynamics, plasticity, reversibility, and memory of its response to cover the full range of functions of activated macrophages [55, 59].

## 5. Advanced Technology for Studying Macrophage Activation

In recent years, with the rapid development of technology, we have been able to analyze the phenotype and function of macrophages though obtained high-resolution data [12]. It helps us reveal the changes in macrophages in health and disease and also provides us with the possibility of different classifications.

### 5.1. The Macrophage Was Analyzed More Deeply and Accurately by Cytometry by Time-of-Flight

Cytometry by time-of-flight is an advanced flow cytometry platform, and it has several technological advancements. When the high-parameter analysis is required, it has advantages over fluorescence-based flow cytometry [60]. The accuracy of CyTOF combined with the mass spectrometric labeling of specific ligands can detect and quantify more than 40 labels at single-cell resolution, and the 135 available detection channels allow the simultaneous study of additional characteristics of complex biological systems across millions of cells [61]. It enables us to have a deeper understanding of the heterogeneity and hierarchical structure of cell population, cell state, multiple signaling pathways, protein hydrolysates, and mRNA transcription [62]. Roussel et al. [63] considered that the monocyte phagocytic system (MPS), including macrophages, was heterogeneous in phenotype and function and used mass cytometry to characterize the deep phenotype of the monocyte phagocytic system. They combined a single mass cytometer panel composed of 38 antibodies with high-dimensional analysis methods to decipher the human MPS compartment in the original sample, associated the results of the primary cells with the *in vitro* marrow exposed to the established polarized inflammatory factors, compared the observation results of human blood and bone marrow cells in lineage differentiation models and established a comprehensive reference frame for the MPS room, and described them using analysis tools such as viSNE, SPADE, and MEM. The results showed that each stimulation condition produced a specific activated macrophage phenotype, with no or almost no overlap between M_IFN-g and M_LPS and M_IL-4 and M_IL-10. It is worth noting that they found different phenotypic patterns in MPS (Table 1).

### 5.2. The Heterogeneity of Macrophages Was Revealed by Single-Cell RNA-seq

**Figure 2:**
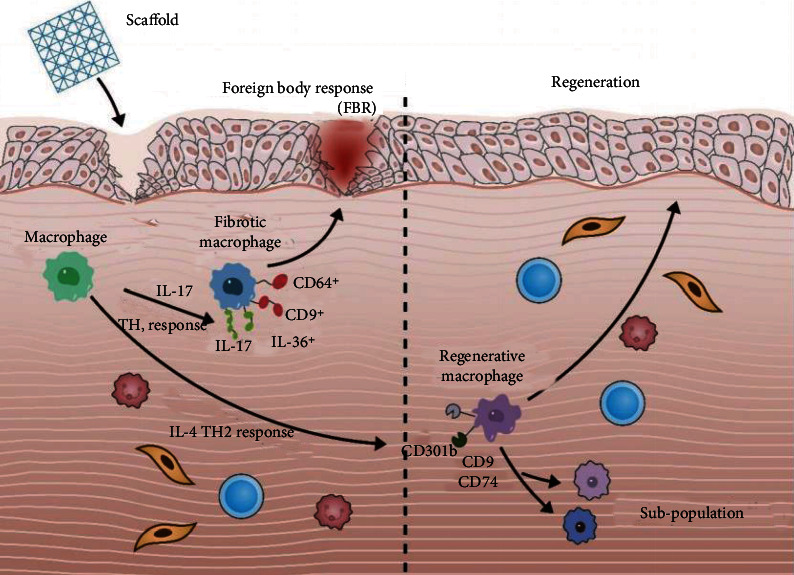
Differentiation of fibrotic macrophages and regenerating macrophages by scRNA-seq. When biological scaffolds are used to repair tissue damage, they can recruit regenerated macrophages through IL-4 and TH2 responses to achieve the purpose of tissue repair, or they can recruit fibrotic cells through IL-17 and TH1 responses to produce foreign body responses. By using scRNA-seq, the study found that CD301b can distinguish regenerated macrophages and can distinguish their subtypes by CD9 and CD74. At the same time, fibrotic macrophages can be distinguished by CD64^+^, CD9^+^, IL-17^+^, and IL-36^+^.

Single-cell RNA-seq can be used to analyze the whole genome and single-cell transcription map of immune cells and it can reveal immune heterogeneity in different diseases. It has become an established method to dissect cell heterogeneity, reveal cell state, and identify the structure of different cell subsets [64, 65]. scRNA-seq can evaluate a large number of genes per cell so that the real population structure can be determined unbiased, and it is possible to identify previously unknown myeloid cell subsets and to understand the dynamic interaction between myeloid cell subsets and other cells of the immune system more quickly [66]. Aran et al. [67] used a scRNA-seq clustering calculation and unbiased annotation tool (Single) to identify macrophages from baseline and mixed lung cell samples after bleomycin-induced mouse lung injury (alveolar macrophages and interstitial macrophages) and applied the hierarchical clustering method to the subgroup of macrophages in fibrosis. The results showed that monocyte-derived disease-related macrophages transformed into alveoli, located in the fibrotic niche, and played a role in promoting fibrosis, and the migration and proliferation of fibroblasts depended on SiglecF+CD11c+MHCIIhi and CX3CR1+ cells secreting Pdgf-aa, indicating that the paracrine interaction between these macrophages and fibroblasts maintains fibroblast proliferation and tissue fibrosis. Some studies have identified a fourth group of cardiac macrophages in the uninjured myocardium through scRNA-seq, and the number of these cells will increase after injury [68]. This population is characterized by a strong interferon-stimulated gene signature called ISG MF. However, it is currently unclear whether ISG macrophages represent a unique subset of tissue macrophages or a part of the activation spectrum, and their role in homeostasis is also unknown. This emphasizes the need for researchers to develop new tools to isolate and explore this population [69]. Sommerfeld et al. [70] used scRNA-seq to describe the relationship between macrophages isolated from mouse tissue repair models and tissue environmental fibrosis after the use of model biomaterials. They used an unbiased clustering algorithm to reveal the diversity of macrophages, calculated and analyzed the phenotypic characteristics of macrophage clusters, defined phenotypic and functional macrophage populations, identified macrophage surface markers by flow cytometry and immunofluorescence techniques, and identified new CD9hi+IL-36 *γ* + macrophage populations. It was found that it had the characteristics of type 17 immune response and autoimmunity and verified the ability to use surface markers to distinguish macrophage subsets.  Figure 2 is shown as follows.

### 5.3. Hyperspectral Images Were Used to Detect and Classify Macrophages in an Unmarked and Noninvasive Manner

The hyperspectral image is an unlabeled and noninvasive way to detect and classify living cells and has significant thermal potential. When it is applied to tissue diagnosis, the resulting three-dimensional data hypercube can encode the properties of light-tissue interaction, such as absorption, scattering, and fluorescence [71]. Based on the spectral characteristics of different tissues, HSI can provide quantitative diagnostic information about histopathology, morphology, and chemical composition of noncontact, noninvasive, and nonionized tissues [72]. Bertani et al. [73] studied human monocyte-derived macrophages by hyperspectral reflectance confocal microscopy and analyzed M1 and M2 activation of hyperspectral data sets by principal component analysis. Then, linear discriminant analysis was used to process HSI data and semiautomatically classify macrophage activation, which confirmed the possibility of single-cell level classification of M1 and M2 macrophages in a noninvasive and unlabeled manner.

## 6. Conclusion

Macrophages are myeloid immune cells, which can be found in almost every tissue of the human body [74, 75]. Their main functions are to participate in host defense, maintain the stability of the tissue environment, remove cell debris, recover apoptotic cells, help tissue regeneration and reparation by secreting cytokines and growth factors, and secrete some proteins, such as extracellular matrix proteins, to take part in cell adhesion [76–81]. According to the activation of macrophages by *in vitro* signals, the classical polarization model divides macrophages into two states. When they are activated as proinflammatory phenotypes and release some cytokines, they can mediate the balance between bone salt deposition, osteogenesis, and osteoclast; for example, activated macrophages can mediate periprosthetic inflammation and make an important impact on recruitment and bone resorption [82–86]. Macrophages play an important role in the early tissue healing process of bone implantation of biomaterials [87]. The success of biomaterial-mediated bone formation depends on the effective and timely conversion of the M1 phenotype to the M2 phenotype during the bone healing process, and the prolonged M1 phase may cause fibrous encapsulation and bone regeneration failure [88]. Osseointegration was defined as a direct structural and functional connection between ordered living bone and the surface of a load-carrying implant [89]. Current research believes that osseointegration is a foreign body reaction, which can protect the implant from the tissue by forming a defense response at the interface bone [90]. The presence of oral implants stimulates higher immune participation through complement and macrophage activation, while macrophage activation can affect the tissue surrounding the implant by regulating inflammation and tissue healing [91]. Studies have shown that the surface topographical and chemical signals on the surface of titanium implants can regulate the polarization of macrophages, and macrophages can also promote the homing and osteogenic differentiation of mesenchymal stem cells on the surface of implants by producing a variety of cytokines and growth factors, thereby regulating the healing process [91, 92]. Lee et al. [93] found that the combination of bioactive ion chemistry and the surface morphology of nanoscale titanium can significantly induce the polarization of M2 macrophages of J774.A1 cells and improve the early bone formation ability of oral implants in animal bones in clinical practice. The activated state is the core of the executive function of macrophages, and it is also the key to immunology, disease pathogenesis, and anti-inflammatory [12, 94, 95]. In some inflammatory diseases, transforming the activation state of macrophages has become a treatment [96, 97]. However, in recent years, some studies have proposed different macrophage activation classification criteria and models based on the behavior of macrophages in the disease process, tissue-specific phenotypes, and high-resolution data obtained through advanced technologies [98, 99]. The heterogeneity of cells and different activation models show that the activation of macrophages is not two extreme changes as described in the classic dichotomy but takes on different forms as environmental stimuli change. The improvement of the macrophage activation model also enables the optimization of immune-based therapeutic measures. These alternative activation models will provide the possibility of treating oral diseases in the future.

## Figures and Tables

**Table 1:** Different phenotypic patterns in MPS by CyTOF [63]. This table summarizes the different phenotypic patterns of macrophages discovered by Roussel et al. using CyTOF in MPS.

Phenotype	Express
M_IL-10	CD32, CD14, CCR2, CD163, CD64, and CD33 highly expressed
M_IL-4	CD274 and CD86 highly expressed; CD14, CD32, and CD33 lowly expressed

## Data Availability

No datasets were generated or analyzed during the current study.
